# Guy's Hospital

**Published:** 1831

**Authors:** 


### GUY'S HOSPITAL.

### Hoo Loo.

This unfortunate inhabitant of the celestial empire traversed many thousand leagues of ocean to lay his bones in a foreign clime! It was well observed by the poet of Nature that, it may sometimes be better to bear those ills we have, than fly to others that we know not of. We were quite convinced, and we mentioned our conviction to more than one person, before the operation, that no human being, and least of all an Asiatic, would survive the removal of such a tumour as *Hoo Loo* presented. Nothing could exceed the dexterity and intrepidity with which this terrible operation was performed by Mr. Key—except the fortitude, patience, and heroic courage with which it was borne by the unfortunate Chinese! The melancholy result of the operation has not, of course, altered the opinion which we formed previously to its performance. The farinaceous diet and the phlegmatic temperament of an Asiatic, control, no doubt, the range of inflammation that must supervene on wounds or operations; but the same circumstances limit his power of withstanding the pain of incisions, and the exhaustion of hæmorrhage. The fact, too, of the genital organs being included in the morbid growth, was a fearful and formidable barrier to the success of the operation. The event shewed, and it was doubtless anticipated by the surgeons, that these organs must be sacrificed in the removal of the tumour. It is true that a somewhat similar operation was performed, with a fortunate result, by M. Delpech, as recorded in a former Number of this Journal; but we do not think that the English surgeon had half the chance of success which encouraged the able Professor of Montpellier. It is with deep regret that we put on record the following concise narrative of this terrible operation.

"The face of the patient was then covered, and Mr. Key, taking his station in front of the tumour, commenced the operation. His object, apparently was to make three flaps, of such a form and extent, as would cover the penis and the testes when they should be relieved from the tumour. The first flap was made on the anterior part of the tumour; the others on either side. The former of these would have covered the penis, and the two others (which were made semilunar in figure with the convexities looking outwards) would have accomplished the purpose, when united, of enveloping the testes, and at the same time of forming the integument of the perineum. The first incision was commenced on the right side, just under the abdominal ring, and being carried obliquely inwards for about an inch, was continued so as to form the semilunar cut, from the lowermost point of which, the knife was again carried inwards, in order, apparently, to leave such a small projection of integument as, with a corresponding piece on the opposite side, might serve to go round the root of the penis. This portion having been reserved, the incision was continued onwards for about four inches in a straight line, and was then turned at a right angle, parallel with the operator, forming a line of about two and a half inches in length. A similar cut was then made on the left side, and connected with that on the right by the parallel or transverse cut. The flap thus formed was now dissected from the tumour and laid back upon the abdomen. The operator then proceeded to lay bare the two cords and the penis, a step in the operation which was performed with very great neatness. Sufficient time had now elapsed for the depressing effects of the operation to exhibit themselves, while the penis and testicles had yet to be dissected out. The determination to attempt this arose from its having been ascertained that the sexual inclinations of the man were unimpaired, seminal emissions being occasionally experienced. The delay, however, which so intricate a portion of the operation would have occasioned, now induced Sir Astley Cooper to propose that the genital organs should be sacrificed, and the suggestion was promply acceded to. A temporary ligature was accordingly passed round each of the cords and the penis, and these being divided, the remainder of the operation was pursued solely with a view to the removal of the whole mass. But a period of time elapsed before the conclusion of the operation which must have far exceeded the anticipations even of the most fearful, and by the time the tumour was entirely separated and the exposed parts were closed over, an hour and forty-four minutes had passed. This tremendous protraction was chiefly occasioned by the intervals which were from time to time allowed the patient for recovery from the fits of exhaustion which supervened. Complete syncope occurred twice, and during the whole of the latter steps of the operation he was in a state of fainting. The quantity of blood lost was variously estimated by those who assisted, and though certainly not large, it was the operator's own impression that the hæmorrhage was the immediate cause of death. It would probably be correct to state the loss at twenty-five ounces, although as few as 14 and as many as 30 were named. Of this quantity not more, we should think, than a single ounce was arterial; all the ligatures were quickly applied, and with great dexterity. The number of large veins divided was immense, but only three small arteries, besides the two spermatic, were taken up. Immediately after the removal of the tumour, another fit of syncope—if syncope could be said to be at all incomplete for the last half hour—came on, from which the poor fellow did not for a moment rally. No remedies that were directed to overcome this state of collapse had the slightest effect; warmth and friction of the extremities, warmth to the scrobiculis cordis, the injection of brandy and water into the stomach, and, ultimately, from the suspicion that the loss of blood had been too great, transfusion to the amount of six ounces, taken from the arm of a student—one amongst several who offered to afford blood—were amongst the means resorted to. The heart's action gradually and perceptibly sunk. The patient did breathe after the operation, but that is as much as can be said. Artificial respiration was subsequently, but vainly attempted."*

The tumour weighed 65 pounds when removed, and it appears to have been elephantiasis. Hoo Loo's health and strength seem to have been very little impaired by the enormous growth—a circumstance that adds to our regret that any attempt should have been made to remove it.

